# Environment-dependent microevolution in a Mediterranean pine (*Pinus pinaster* Aiton)

**DOI:** 10.1186/s12862-014-0200-5

**Published:** 2014-09-23

**Authors:** Ricardo Alía, Regina Chambel, Eduardo Notivol, José Climent, Santiago C González-Martínez

**Affiliations:** Department of Forest Ecology and Genetics, INIA-Forest Research Centre (CIFOR), Avda. A Coruña km 7.5, 28040, Madrid, Spain; Sustainable Forest Management Research Institute, University of Valladolid-INIA, Madrid, Spain; Unit of Forest Resources, CITA. Carr. Montañana s/n, 50059 Zaragoza, Spain; Department of Ecology and Evolution, University of Lausanne, 1015, Lausanne, Switzerland

**Keywords:** Adaptive traits, Response to selection, Selection gradients, Phenotypic directional selection, Mediterranean forest

## Abstract

**Background:**

A central question for understanding the evolutionary responses of plant species to rapidly changing environments is the assessment of their potential for short-term (in one or a few generations) genetic change. In our study, we consider the case of *Pinus pinaster* Aiton (maritime pine), a widespread Mediterranean tree, and (i) test, under different experimental conditions (growth chamber and semi-natural), whether higher recruitment in the wild from the most successful mothers is due to better performance of their offspring; and (ii) evaluate genetic change in quantitative traits across generations at two different life stages (mature trees and seedlings) that are known to be under strong selection pressure in forest trees.

**Results:**

Genetic control was high for most traits (*h*^*2*^ = 0.137-0.876) under the milder conditions of the growth chamber, but only for ontogenetic change (0.276), total height (0.415) and survival (0.719) under the more stressful semi-natural conditions. Significant phenotypic selection gradients were found in mature trees for traits related to seed quality (germination rate and number of empty seeds). Moreover, female relative reproductive success was significantly correlated with offspring performance for specific leaf area (SLA) in the growth chamber experiment, and stem mass fraction (SMF) in the experiment under semi-natural conditions, two adaptive traits related to abiotic stress-response in pines. Selection gradients based on genetic covariance of seedling traits and responses to selection at this stage involved traits related to biomass allocation (SMF) and growth (as decomposed by a Gompertz model) or delayed ontogenetic change, depending also on the testing environment.

**Conclusions:**

Despite the evidence of microevolutionary change in adaptive traits in maritime pine, directional or disruptive changes are difficult to predict due to variable selection at different life stages and environments. At mature-tree stages, higher female effective reproductive success can be explained by differences in their production of offspring (due to seed quality) and, to a lesser extent, by seemingly better adapted seedlings. Selection gradients and responses to selection for seedlings also differed across experimental conditions. The distinct processes involved at the two life stages (mature trees or seedlings) together with environment-specific responses advice caution when predicting likely evolutionary responses to environmental change in Mediterranean forest trees.

**Electronic supplementary material:**

The online version of this article (doi:10.1186/s12862-014-0200-5) contains supplementary material, which is available to authorized users.

## Background

To confront the rapid environmental change caused by global warming, plants will have either to migrate or to adapt in-situ to new conditions [1]. Mounting evidence on seed dispersal and plant migration rates has revealed that plant migration capability falls short, in various orders of magnitude, of that needed to track ecological optima in the near future [2], thus highlighting the importance of within-population standing genetic variation, phenotypic plasticity, and adaptive evolution for plant population survival. In the case of foundation species such as forest trees (see [3]), their adaptive potential is of paramount importance, as it allows to predict the evolutionary responses of entire terrestrial ecosystems in the context of global climate change [4]. A central question in the adaptability of forest tree species to changing environments is their potential for short-term genetic change resulting from differences in parental reproductive success and selection at early recruitment stages, when mortality rates are higher.

Adaptive evolutionary change at any given trait requires an association between the trait and fitness, plus the heritable transmission of the trait across generations [5]. The first component can be estimated by phenotypic and genetic selection gradients, and the second by measuring the response to selection. Phenotypic selection gradients (defined as the vector of partial regression coefficients of individual relative fitness on the traits [6]) have been characterized for different plant species at population level, especially through parentage analysis based on molecular markers (e.g. [7,8]). They pose, however, some limitations derived from (i) potential trait multicollinearity, (ii) biases introduced by failure to include traits that covary with fitness, (iii) deviations of response variables from multivariate normality assumed for hypothesis testing, and (iv) failure to consider environmental factors that may induce spurious correlations [9]. Furthermore, it is not clear what the implications are of such selection gradients for a population’s adaptive potential, i.e. its ability to respond to selective pressures [10]. Some of the limitations mentioned can be overcome by using genetic selection gradients (i.e. the strength of selection acting on breeding values), as breeding values “are correlated with their corresponding phenotypic value but are, by definition, uncorrelated with error deviations for fitness as long as there is no genetic correlation between the measured traits and the unmeasured, intervening phenotypic character” [9]. The second component of adaptive evolutionary change, the evolutionary response to selection (represented by *R*), depends on genetic variation (i.e. heritability) and the selection differential (breeders’ equation) of the trait considered. This component is difficult to estimate, especially for long-lived organisms in natural conditions ([11]; see [12] for some estimates in different organisms and the problems involved).

In ecological and evolutionary studies, fruit set has often been considered a convenient proxy of fitness (e.g. [13]). Common observation of a large variance of reproductive success (or relative fertility) in natural populations (e.g. [14]) supports a relevant role of fecundity in short-term evolutionary change. Indeed, the mass effect created by some parents’ higher reproductive output, through larger fruit and seed crops, could lead to higher recruitment of their offspring [15] and, if heritable genetic differences exist for adaptive traits among parent trees, then short-term change in population means is expected for those traits. However, fruit and seed production may have limited value as predictors of genetic change across generations because of differences in seed (e.g. less inbred seed) or offspring (in terms of performance) quality and typically strong post-dispersal selection [16-20]. In long-lived organisms, such as forest trees, the seedling stage is a particularly critical part of the life cycle because seedlings are more susceptible to resource limitation, which affects establishment and early growth [21], and suffer from extremely high mortality rates (up to 90% during the first growing season [22,23]). Seedlings are thus expected to be under strong selective pressure in natural conditions [24]. Success in recruitment, then, results from a complex combination of abiotic and biotic factors that are spatially variable, which reduces the utility of fruit set as fitness proxy and highlights the need to include seedling performance under contrasted environments in evolutionary change studies. This is particularly true in highly heterogeneous landscapes such as the Mediterranean or tropical forests [20].

In this study, we used *Pinus pinaster* Aiton (maritime or cluster pine) as experimental model species to study short-term evolutionary change under contrasted environmental conditions. Maritime pine is a long-lived organism (up to ~200 years under natural conditions) with a large distribution range and important ecological and economic value in the western Mediterranean basin. Large differences in effective reproductive success and molecular-based (phenotypic) selection gradients for different mother traits (as in [8], but considering successfully established regeneration) have been reported in a typical Mediterranean natural population (Coca, Spain) using neighborhood mating models [25,26]. However, offspring performance of the most successful mothers and, thus, the role of observed differences in female reproductive success on short-term genetic change is unknown.

The objectives of our study are i) to test whether higher recruitment of the most successful mothers is due to better performance of their offspring or, alternatively, simply a result of the mass effect of higher female fertility, and ii) to evaluate genetic changes in quantitative traits across generations at different life stages (mature trees and seedlings) and their expected role for adaptability to future environmental conditions. We addressed these goals by estimating breeding values of mother trees with contrasted effective (i.e. based on successful regeneration) reproductive success via progeny testing under controlled and semi-natural conditions. Our experiment protocols allowed us to hasten ontogenetic change and decompose growth into different components that have a high genetic control [27-29]. As detectable response to selection may depend on experimental conditions, we tested progenies under different levels of water availability and intra-specific competition, two highly-relevant selective drivers in Mediterranean maritime pine forests. Finally, short-term genetic change was evaluated by estimating phenotypic (mature trees) and genetic (seedlings) selection gradients and response to selection for a wide range of adaptive traits.

## Methods

### Plant material and seed collection

We sampled 22 mature trees (see below for details on tree selection) from a natural maritime pine stand located in a continuous forest in Central Spain (Coca, Segovia). No forest management has been applied to this area in the last 40 years except for removal of dead trees. Mature, well-formed cones were collected from each tree for progeny testing. The cones were collected for the same year, to avoid interannual environmental effects, and for each mother tree separately. Thus, each seed lot had an approximate structure of half-sib families (since correlated paternity within maternal progenies is known to be negligible in maritime pine, see [30]). Seeds were extracted from several cones from each mother and stored in dry conditions at 4°C.

### Reproductive success and field measurements

Twenty-two mother trees were selected to cover the variation in female reproductive success found in the stand. Previously [26], a study on gene dispersal and female reproductive success based on parentage analysis of naturally-regenerated saplings using nuclear microsatellite (nuSSR) molecular markers had been conducted. This study included 380 mature trees sampled in an area of 3.2 ha and 267 naturally-established saplings, considered the basis for next generation’s population, collected from a central subplot of 30 m in radius. Female parentage LOD scores were retrieved from this study. Briefly, the most-likely parents and parent pairs were detected using log-likelihood ratios (LOD-scores) [31,32] and population allele frequencies estimated from the whole dataset (n = 581). Only highly reliable parent-offspring matches (LOD-score > 4.4, as shown by simulation) were used (see details in [32,33]). Following González-Martínez et al. [26], the parent closer to the offspring was considered to be the mother in parent pairs, also because pines present a much more restricted seed than pollen dispersal. LOD scores for each mother-offspring relationship were summed across all offspring for each mother and then divided by the sum of LOD scores across all mothers, producing a relative measure of female reproductive success for each mother tree. Estimates of reproductive success based on fractional paternity assignment are normally less biased than those relying on direct parent-offspring identification [34]. To remove biases due to distance effects, individual reproductive success values were corrected by the probability of a propagule dispersed from the focal female landing within the central subplot where saplings were sampled (assumed for mathematical convenience to be a square of equal area than the actual central subplot). This probability was calculated via numerical integration of a bivariate exponential-power dispersal kernel with parameters *α* = 68.6 and *β* = 3, which are the best out of three inverse-modeling dispersal kernels for saplings fitted in [26].

For each of the 22 mother-trees with contrasting values of relative reproductive success (see Additional file 1: Table S1 in Supplementary Information for a summary), we measured different fitness-related traits: size (measured by total height –HT– and diameter –DBH–), diameter increment in the last 10 years (DBH10), number of cones, seed weight (based on a 100-seed sample), germination rate (after 35 days), and number of empty seeds (in a 100-seed sample).

### Experimental design for progeny testing

To estimate breeding values, two different experiments were conducted using progenies from mother trees with contrasting effective reproductive success. The first one (*Experiment 1*) was a growth-chamber experiment under environment-controlled conditions, the second (*Experiment 2*), an outdoor experiment under semi-natural conditions. Individual identification of the seeds and seedlings was maintained for the duration of the experiments.

#### Experiment 1: Environment-controlled conditions

A sample of 100 seeds from each of 18 mother trees (see Additional file 1: Table S1 in Supplementary Information) were weighed and germinated under controlled conditions (20°C, 12 h light) using a sand-substrate. After germination, 24 seedlings per family were transplanted to individual 400 cc pots, with a sand-peat (2:1) substrate, and were moved to a growth chamber under controlled conditions. Prior to transplanting, each pot, including the substrate, was weighed (dry weight and full capacity weight) to determine water content at full capacity. The plants were arranged in four blocks (two for each watering regime, see below) and a row-column design (within blocks), with six experimental units (one-seedling plot) for each genetic entry, hence totaling 24 seedlings per family. The conditions of the experiment were set to hasten ontogenetic changes [27], aiming at maximizing developmental differences in a short cultivation time. This, in addition, helped to control root restriction effects due to container to a minimum [35]. The experimental phase was divided in three (acclimation, growing –including the watering treatment period–, and hardening), depending on temperature and photoperiod (see Additional file 1: Table S2 in Supplementary Information). The first watering regime consisted on keeping plants at full-field capacity (W1, 45% in volume), and the second on reducing water availability to 20% of full-field capacity (W2, 10% in volume). The two watering regimes were controlled by weighing two pots per family and block every second day. In total, the experiment lasted for 189 days (84 days under watering treatments).

#### Experiment 2: Outdoor semi-natural conditions

Twenty-four seeds from each of 21 mother trees (see Additional file 1: Table S1 in Supplementary Information) were weighed and germinated under controlled conditions (20°C, 12 h light) using a sand substrate and transplanted to outdoor semi-natural conditions. Transplanting was done periodically by blocks, in order to keep the differences due to germination included in the block effect. The experimental site was located in Madrid (40° 27’ 23.82” N, 3° 45’ 6.91” W, 597 m of altitude) under average environmental conditions similar to those of the seed source stand (rainfall: 452 mm, summer precipitation: 58 mm, temperature: 14.4°C, mean temperature of the coldest month: 5.3°C, mean temperature of the warmest month: 24.8°C). Actual climatic conditions during the outdoor experiment were extremely dry but similar to those in which the previous generation (i.e. the mother trees) of the population studied underwent natural selection (period 1903–1942, data not shown). As in the previous experiment, the plants were arranged in four blocks and a row-column design (within blocks), with six experimental units (one-seedling plot) for each genetic entry (24 seedlings per family). We used two contrasting planting densities; two blocks were planted at a 10x10 cm spacing (D1, 100 plants/m^2^), and the other two at 5x5 cm (D2, 400 plants/m^2^), to elicit contrasting inter-family competition effects. Seedlings were protected to avoid predation by animals (rodents, birds, or ants), and no special management was applied during the experiment duration. The experiment lasted 203 days, from April 12^th^ to November 1^st^.

#### Phenotype measurements in seedlings

Phenotypic traits were chosen for their functional and adaptive importance in pines under limiting resource supply: growth and biomass allocation [36-38], specific leaf area [39] and ontogenetic change [40]. These traits present large differences among maritime pine families and populations evaluated in controlled conditions [41-43]. In addition, most of them seemed to have undergone adaptive genetic differentiation (as evaluated by *Q*_*st*_) in response to local climatic conditions, when tested in rangewide experiments [44].

For the two experiments, total height and shoot developmental status were evaluated in all seedlings every second week, the latter following a subjective scale based on the type of axillary shoots, as described in detail in [42]. The height of the plant at ontogenetic stage 3 (HON3) –corresponding to the earliest observation of axillary long shoots– was used for subsequent analyses as a size-independent quantitative index of seedling development. Higher values of HON3 indicate a slower seedling ontogenetic development. Height growth of each seedling was decomposed by adjusting a Gompertz model [29] and analyzing the three main parameters of this curve: HTOT, *b*, and *m*. HTOT is the total height at the end of the experiment (asymptote), the *b*-parameter is related to the maximum growth rate standardized by HTOT, and *m*, the inflection point, indicates whether maximum growth is reached at the beginning (low values of *m*) or the end (high values of *m*) of the growing period.

At the end of each experiment, survival (SUR) was recorded, all plants were harvested, and dry mass of each fraction (needles, stem, and roots) was determined with 0.001 g precision. The dry mass of each fraction was expressed relative to that of the total plant (leaf mass fraction, LMF; stem mass fraction, SMF; root mass fraction, RMF) [36]. Total dry mass (DW) was obtained as the sum of leaf, stem, and root dry mass. Specific leaf area (SLA) was estimated from 10 needles randomly chosen from each plant. Finally, since at the seedling stage every metamer bears a primary needle, the number of stem units (NSU) was estimated by comparing total primary needle dry mass to dry mass of the 10 needles collected for SLA assessment.

#### Quantitative genetic models in progeny tests

For each variable and experiment, a mixed model was adjusted using Restricted Maximum Likelihood (REML):

$$ {y}_{ijkl}=\mu +{\varphi}_i+{T}_j+{\chi}_{ij}+B{(T)}_{k(j)}+c+{\varepsilon}_{ijkl} $$

where *y*_*ijkl*_ is the phenotypic value of the variable for the *l*^*th*^ tree from the *i*^*th*^ family in the *j*^*th*^ treatment located in the *k*^*th*^ block, *μ* is the overall mean, *ϕ*_*i*_ is the effect of the *i*^*th*^-family (*i* varying from 1 to 18–21, depending on the experiment), *T*_*j*_ is the effect of the *j*^*th*^ treatment (two watering levels in the growth chamber experiment and two density levels in the outdoor experiment, thus *j* varying from 1 to 2 in each experiment), *χ*_*ij*_ is the family by treatment interaction, *B*_*k*_ is the effect of the *k*^*th*^-block (1–4), *c* is the covariate effect, and *ε*_*ijkl*_ the residual. For survival, a generalized logistic mixed model (GLMM) was applied with a binomial distribution and a logit link function. For biomass traits (LMF, SMF, RMF), the total dry mass (DW) was used as a covariate to correct for allometric changes [36]. For the rest of the quantitative traits, seed weight (WSEED) was used as a covariate to control for maternal effects. Family and treatment by family interaction were considered random factors. The models were fitted using ASREML [45].

#### Genetic control

Narrow-sense heritability (*h*^*2*^) was estimated for each trait and progeny test following standard procedures [46]. For survival (SUR), the residual variance of the GLMM was set to π^2^/3 [47]. The standard error of heritability was estimated using a Taylor’s series approximation [45]. Best Linear Unbiased Predictors (BLUPs) are weighted means commonly used as point estimates of random effects in mixed models. They predict the expected phenotype of a tree’s offspring or an individual by using phenotypic information collected from relatives, which is useful to evaluate genetic changes in the populations after selection. Mother-tree breeding values (based on offspring performance) for each trait were computed from the heritability of the trait and the BLUPs estimated in the experiments [45,46]. Evolvability, as estimated by the coefficient of additive genetic variation (CV_A_), was also computed [10].

### Phenotypic selection gradients and correlation of female reproductive success with offspring performance

Phenotypic selection gradients were computed based on traits measured directly on the mother-trees in the field, to evaluate the relationship among trait values in adults and their fitness (in terms of relative reproductive success), as obtained from molecular marker-based parentage analysis (see [26] and above). The proxy for fitness of parent trees used in this study (i.e. the actual number of successful naturally-regenerated offspring) is probably more accurate than amount of pollen donated, flower production or fruit set (e.g. [8,13]), as the latter three do not consider post-dispersal mortality and early selection. These selection gradients were computed as the vector of partial regression coefficients of individual relative reproductive success on trait values. Given the reduced number of mother trees in the study (22), we performed a Principal Component analysis to reduce the number of dimensions in the analysis. The following log-linear model was used ([7] and references therein) to define female relative reproductive success (*λ*_*k*_):

$$ Ln\left({\lambda}_k\right)={\beta}_1{z}_{1k}+{\beta}_2{z}_{2k}+{\beta}_3{z}_{3k}+{\gamma}_1{z}_{1k}^2+{\gamma}_2{z}_{2k}^2+{\gamma}_3{z}_{3k}^2 $$

The subscripts (1 to 3) refer to each of the three first principal components (explaining 69.64% of the variance, see Additional file 1: Table S4 in Supplementary Information). In biological terms, the *β*_*i*_ are analogous to linear selection gradients, and the *γ*_*i*_ are analogous to quadratic selection gradients as defined by Lande and Arnold [6]. Traits were standardized, and individual fitness was converted to relative fitness by dividing by the mean fitness of all individuals within the subset of data. The statistical significance of each specific selection gradient, *β*_*i*_ or *γ*_*i*_, was estimated using likelihood-ratio tests, by subtracting the log-likelihood for the model excluding each parameter, one at a time, from the log-likelihood of the full model, which is asymptotically chi-square distributed with one degree of freedom.

Finally, to know to what extent mother reproductive success (and the associated phenotypes) is driving population phenotypic change across generations, we computed the relationship between female reproductive success and breeding values for each trait, as obtained from offspring performance under the two experimental environments, using log-linear models. Insights into this relationship are especially relevant at early establishment stages, when natural selection is stronger.

### Genetic selection gradients and response to selection at early stages of establishment

Genetic selection gradients at seedling stage were computed in the two experiments using seedling height (HTOT), which is related to competitive ability and crucial for natural regeneration establishment in pines [48], and/or survival (SURV) as fitness proxies. Both fitness proxies were used in the outdoors semi-natural experiment while only seedling height was used to gauge fitness under the optimal growing conditions of the controlled-environment experiment, as no significant genetic differences among families were found for survival in this experiment (see Results). Genetic selection gradients were obtained by bivariate analysis (to reduce the bias in the estimation, see [9,12]), as the genetic covariance between the trait and fitness divided by the genetic variance of the trait, for the traits estimated in the two experiments. In addition, we computed the genetic response to selection (*R*), following the Price Theorem [5], as the covariance between the trait and fitness. Standard errors were computed using a Taylor’s series approximation [45].

## Results

### Genetic control, and interactions and main effects of water regime and density

Seedlings under semi-natural conditions almost doubled the specific leaf area (SLA) of those under controlled conditions (*Experiment 1*), and also had higher height and biomass (with a higher allocation to the stem fraction), but the development of axillary shoots (i.e. the same ontogenetic stage) was reached at a similar size (HON3). Genetic control varied depending on the trait and experiment (Table 1). In the growth chamber experiment (*Experiment 1*), narrow-sense heritability (*h*^*2*^) was significantly different from zero for all traits except for *b*, the standardized maximum growth rate (obtained from decomposition of each seedling height growth using a Gompertz model), and survival (SUR). Under semi-natural conditions (*Experiment 2*), genetic control was much lower except for maturation (HON3), total height growth (HTOT) and survival (SUR). Strong genetic control of HTOT and SUR was somehow expected, as these two parameters are directly associated with fitness at early stages of establishment in forest trees. A positive correlation (*r* = 0.694) was observed between HTOT and SUR breeding values under growth chamber conditions, but this correlation was strongly negative (*r* = −0.637) under semi-natural ones (see Additional file 1: Table S3 in Supplementary Information). Finally, genetic values were correlated across experiments only for HTOT (positive, *r* = 0.418) and SLA (negative, *r* = −0.478).

**Table 1:** **Genetic control for adaptive traits in maritime pine under two contrasted experimental conditions**

**Trait**	**Environment**-**controlled conditions** **(** ***Experiment 1*** **)**				**Outdoor semi**-**natural conditions** **(** ***Experiment 2*** **)**			
	***h*** ^***2***^	**S.E.(** ***h*** ^***2***^ **)**	**MEAN**	**CV**_**A**_	***h*** ^***2***^	**S.E.(** ***h*** ^***2***^ **)**	**MEAN**	**CV**_**A**_
LMF	0.350	0.163	0.541	3.4	0.068	0.072	0.469	5.3
SMF	0.357	0.171	0.055	6.3	0.041	0.064	0.163	12.0
RMF	0.332	0.158	0.403	4.8	0.137	0.091	0.367	5.4
DW	0.377	0.170	0.892	5.5	0.110	0.083	0.485	29.1
SLA	0.253	0.162	57.035	2.5	0.071	0.073	82.5	4.7
NSU	0.611	0.246	37.287	8.4	0.066	0.071	85.8	19.6
HON3	0.137	0.103	24.951	1.9	0.276	0.126	27.48	9.7
*b*	0.000	NA	NA	NA	NA	NA	NA	NA
*m*	0.297	0.157	36.328	0.9	NA	NA	NA	NA
HTOT	0.876	0.2785	19.231	8.8	0.415	0.159	46.94	13.1
SUR	0.226	0.2300	0.767	NA	0.719	0.243	0.715	NA

*LMF*: leaf mass fraction; *SMF*: stem mass fraction, *RMF*: root mass fraction; *DW*: dry weight; *SLA*: specific leaf area; *NSU*: number of stem units; HON3: height at first production of axillary shoots; *b*, *m*: coefficients of the Gompertz function; HTOT: total height; *SUR*: Survival; *NA*: not available.

*h* ^*2*^: heritability; S.E. (*h* ^*2*^): standard error of *h* ^*2*^; MEAN: mean value of the population; CV_A_: coefficient of additive genetic variation.

Heritability estimates are shown only when family effects were statistically significant.

In both experiments and for all traits, the family per treatment (water regime in *Experiment 1* and density in *Experiment 2*) interaction term had very little importance (less than 5% of the total variance; Table 2). The lack of family per treatment interaction for survival and other traits related to fitness at early life stages, such as total height (HTOT), indicated reduced opportunities for family specialization caused either by intraspecific competition or by watering regime under our experimental conditions. With respect to main effects of treatments, a highly significant effect of the watering regimes was found in *Experiment 1* for all traits except for maximum height growth rate (parameter *b* of the Gompertz function) (Table 2). The covariates, which accounted for allometric changes (dry weight, DW) and maternal effects (seed weight, WSEED) in this experiment, were also highly significant for most traits (except for stem mass fraction, SMF, specific leaf area, SLA, and ontogenetic change, HON3). Under the semi-natural outdoor conditions (*Experiment 2*), seeding density had little influence on biomass allocation traits, survival (SUR) and number of stem units (NSU), but high effects in dry weight (DW), specific leaf area (SLA), ontogenetic change (HON3) and height (HTOT). The mixed effects of density, i.e. intraspecific competition, on adaptive traits highlight the need to evaluate various components of fitness, in this case SUR and HTOT, in microevolutionary studies. As for Experiment 1, the covariates, dry weight (DW) and seed weight (WSEED), were highly significant for most traits (except for specific leaf area, SLA).

**Table 2:** **Mixed model analysis of two experiments** (**under controlled and semi**-**natural conditions**) **in maritime pine**, **including fixed effects** (***F***-**value and significance level**) **and covariates** (**DW**: **dry weight**, **WSEED**: **seed weight**)

**Trait**	**Environment**-**controlled conditions** **(** ***Experiment 1*** **)**						**Outdoor semi**-**natural conditions** **(** ***Experiment 2*** **)**						
	***F***-**value**, **significance level** ^**1**^			**%Var**	**Mean** ^**2**^		***F***-**value**, **significance level** ^**1**^			**%Var**	**Mean** ^**2**^		
	**TRT**	**BLOCK**	**COV**	TRT*FAM	**W1**	**W2**	**TRT**	**BLOCK**	**COV**	TRT*FAM	**D1**	**D2**	**Covariate**
LMF	28.70 ***	7.43 **	20.19 ***	0.0 ns	0.431	0.486	0.04 ns	12.14 ***	58.01 ***	1.4 ns	0.477	0.461	DW
SMF	9.00 **	1.81 ns	0.61 ns	3.7 ns	0.054	0.059	0.01 ns	8.71 **	24.73 ***	0.0 ns	0.161	0.165	DW
RMF	35.24 ***	7.96 **	16.16 ***	0.0 ns	0.514	0.454	0.03 ns	10.85 ***	19.60 ***	1.0 ns	0.361	0.379	DW
DW	280.23 ***	62.17 ***	29.62 ***	0.0 ns	0.7406	1.0519	7.13 **	4.69 **	9.08 **	0.0 ns	0.4257	0.5725	WSEED
SLA	77.76 ***	26.80 ***	1.21 ns	0.0 ns	49.59	56.70	12.26 ***	3.67 *	1.72 ns	2.8 ns	84.99	79.16	WSEED
NSU	40.56 ***	40.90 ***	15.82 **	3.8 ns	36.53	45.30	2.27 ns	1.99 ns	9.71 **	0.0 ns	82.13	90.88	WSEED
HON3	7.73 **	0.28 ns	3.16 ns	2.6 ns	25.91	27.61	21.80 ***	6.60 **	9.10 **	0.0 ns	26.07	29.45	WSEED
*b*	0.03 ns	1.06 ns	5.22 *	0.0 ns	0.0637	0.0663	NA	NA	NA	NA	NA	NA	WSSED
*m*	101.79 ***	3.57 *	10.59 **	2.6 ns	191.3	204.2	NA	NA	NA	NA	NA	NA	WSEED
HTOT	159.48 ***	4.48 *	5.01 *	0.0 ns	34.47	42.45	8.73 **	2.55 ns	16.60 ***	0.0 ns	44.82	50.31	WSEED
SUR	14.49 **	7.11 **		4.4 ns	0.827	0.594	0.08 ns	1.22 ns		0.0 ns	0.722	0.697	

*TRT*: treatment (W1/W2, D1/D2; see Methods); *COV*: covariate;%Var: Percent of the interaction term (TRT*FAM) with respect to the total variance; *LMF*: leaf mass fraction; *SMF*: stem mass fraction; *RMF*: root mass fraction; *DW*: dry weight; *SLA*: specific leaf area; *NSU*: number of stem units; *HON*3: height at first production of axillary shoots; *b*, *m*: coefficients of the Gompertz function; *HTOT*: total height; *SUR*: survival; *NA*: not available.

^1^ns: not significant, *: 0.05 ≤ α < 0.01, **: 0.01 ≤ α < 0.001, ***: α ≤ 0.001.

^2^Best linear unbiased estimates (BLUE) for watering (W1 and W2) and density (D1 and D2).

### Phenotypic selection gradients and correlation of female reproductive success with offspring performance

Relative effective reproductive success in natural conditions (i.e. based on successfully established offspring) varied one order of magnitude (0.0078-0.0942) among mother trees (as retrieved from a previous study, see [26]). Selection gradients based on mother-tree phenotypic traits rely on direct field measurements (Table 3). Only the linear phenotypic selection gradient for the PC2 (related to germination rate and number of empty seeds, i.e. seed quality) was significant, with the quadratic selection gradient for this principal component being marginally significant (*P* = 0.0674).

**Table 3:** **Phenotypic selection gradients for maritime pine based on mother**-**tree traits**

**Trait**	**DF**	**Selection gradient**	**Standard Error**	**Wald 95%** **CIs**		**Chi**- **Square**	**Pr > ChiSq**
*Linear selection gradient*							
PC1	1	0.2140	0.1366	−0.0538	0.4818	2.45	0.1173
*PC2*	*1*	*0.4313*	*0.2045*	*0.0306*	*0.8321*	*4.45*	*0.0349*
PC3	1	−0.0289	0.1247	−0.2733	0.2156	0.05	0.8170
*Quadratic selection gradient*							
PC1^2^	1	−0.0073	0.0802	−0.1645	0.1499	0.01	0.9273
PC2^2^	1	0.1090	0.0596	−0.0078	0.2259	3.35	0.0674
PC3^2^	1	−0.1609	0.0974	−0.3518	0.0301	2.73	0.0987

Significant selection gradients are given in italics (Additional file 1: Table S4 for details of the PC analysis).

PC1 main loading factors: age (0.813), height (0.798) and diameter (0.790); PC2 main loading factors: number of empty seeds (−0.768) and germination rate (0.896); PC3 main loading factors: seed weight (0.894).

**Figure 1:**
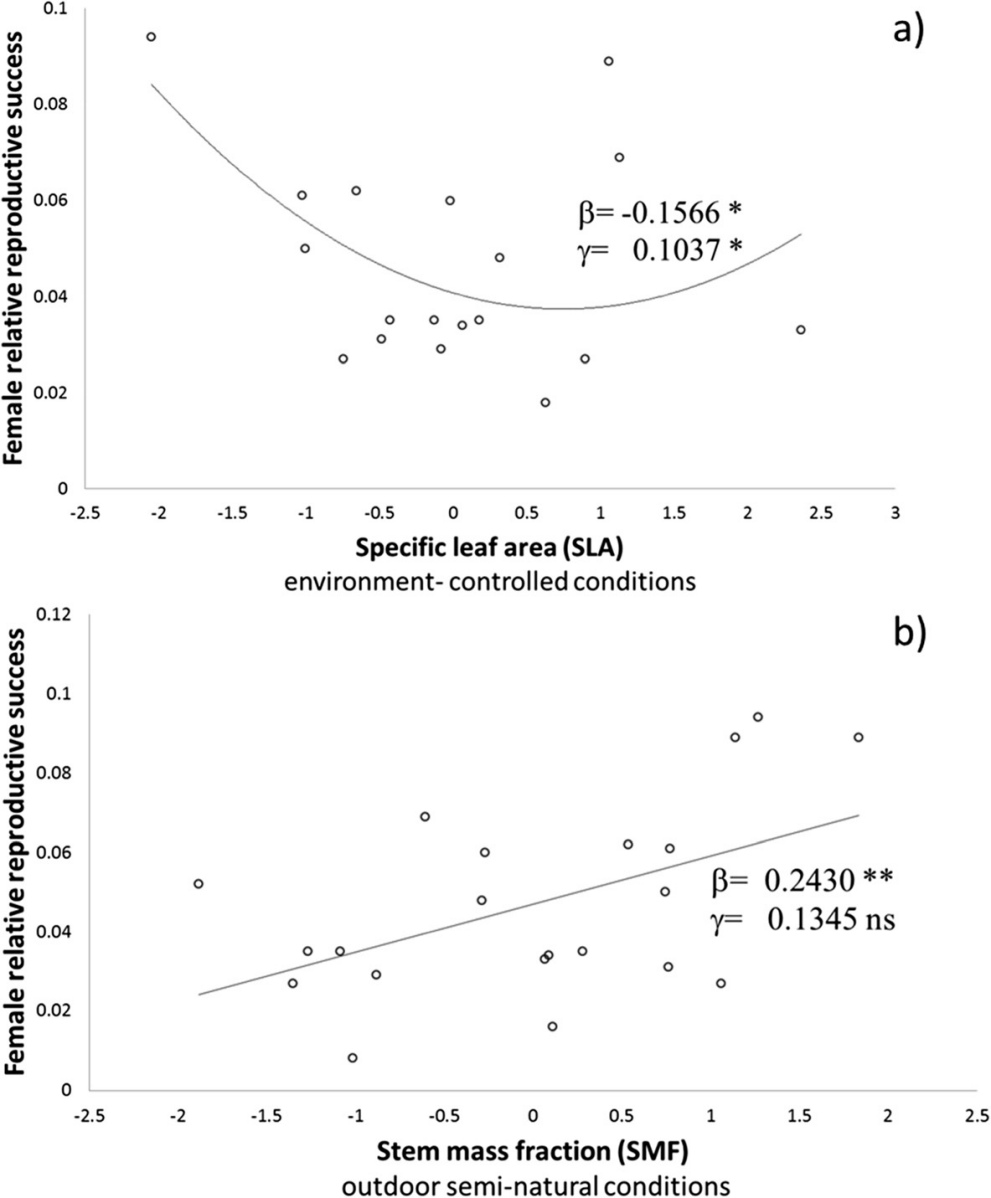
**Relationship between female relative reproductive success and breeding values for a) Specific leaf area (SLA) and b) Stem mass fraction (SMF) measured in environment-controlled conditions (** ***Experiment 1*** **) and outdoor semi-natural conditions (** ***Experiment 2*** **), respectively.** Parameters and significance values corresponding to the log-linear model are included; ns: not significant, *: 0.05 ≤ α < 0.01, **: 0.01 ≤ α < 0.001.

We found significant linear correlations (of different sign) between female reproductive success and offspring performance (as estimated by breeding values) in two traits: negative for specific leaf area (SLA) in *Experiment 1* and positive for stem mass fraction (SMF) in *Experiment 2* (Figure 1). The quadratic term of the log-linear model was also significant for the former. These results, together with contrasted heritability across experiments, illustrate distinct expected microevolutionary changes due to differences in female reproductive success depending on environmental conditions.

### Genetic selection gradients and response to selection at early stages of establishment

Genetic selection gradients were computed at early establishment stages based on genetic covariance of offspring traits with total height (in both experiments) and survival (only in the outdoor experiment, *Experiment 2*, as this trait was not under genetic control in *Experiment 1*; see above) as fitness proxies (see Methods). When considering total height, selection gradients were significant for stem mass fraction (SMF, positive) and the inflection point of the growth curve (parameter *m*, negative; i.e. plants that reached maximum growth at the beginning of the growing period showed higher fitness) under environment-controlled conditions (*Experiment 1*), and for the ontogeny index (HON3, positive; i.e. plants with slower ontogenetic development showed higher fitness) under outdoor experimental conditions (*Experiment 2*) (Table 4). Response to selection (*R*) for these variables ranged (in absolute values) from 0.08 (*m*) to 0.12 (SMF and HON3). When survival (SUR) was considered as a fitness proxy in the outdoor experimental conditions (*Experiment 2*), the results were similar, with HON3 being the only trait with a significant (and positive) effect on fitness.

**Table 4:** **Genetic selection gradients for offspring** (**i.e. based on genetic covariance of traits with seedling height** –**HTOT**– **and**/**or survival** –**SUR**– **as proxies of fitness**; **see** **Methods**) **and response to selection** (***R***) **in two experimental conditions in maritime pine**

**Trait**	**Environment controlled conditions** **(** ***Experiment 1*** **)**				**Outdoor semi**-**natural conditions** **(** ***Experiment 2*** **)**							
	**HTOT**				**HTOT**				**SUR**			
	**Selection gradient**	**S.E.**	***R***	**S.E.**	**Selection gradient**	**S.E.**	***R***	**S.E.**	**Selection gradient**	**S.E.**	**R**	**S.E.**
LMF	0.2466	0.4444	0.020	0.037	NE	NE	0	0	NE	NE	NE	NE
*SMF*	*1.4059*	*0.3697*	*0.121*	*0.048*	−1.2667	5.3920	−0.008	0.027	2.1487	12.2429	0.012	0.062
RMF	−0.6386	0.4441	−0.048	0.038	0.3422	1.4835	0.007	0.029	−2.0826	2.9119	−0.052	0.069
DW	1.0307	0.5029	0.048	0.030	NE	NE	NE	NE	3.3621	1.1801	0.289	0.104
SLA	0.8369	0.7142	0.039	0.033	NE	NE	NE	NE	NE	NE	NE	NE
NSU	0.7501	0.3275	0.078	0.045	NE	NE	NE	NE	4.3425	2.2636	0.217	0.089
*HON3*	1.5480	0.898	0.052	0.033	*1.0725*	*0.2516*	*0.120*	*0.052*	*1.8632*	*0.6731*	*0.229*	*0.119*
*m*	−*1.5200*	*0.5959*	−*0.082*	*0.036*	NE	NE	NE	NE	NE	NE	NE	NE

*LMF*: leaf mass fraction; *SMF*: stem mass fraction, *RMF*: root mass fraction; *DW*: dry weight; *SLA*: specific leaf area; *NSU*: number of stem units; *HON*3: height at first production of axillary shoots; *m*: the inflection point of the growth curve.

*S.E*. standard error of the estimates. Significant values are given in italics; *NE*: not estimable.

## Discussion

This paper explores the potential for micro-evolutionary changes in a natural population of *Pinus pinaster* at two different stages of development: mature trees and seedlings. To our knowledge, there are no studies on the estimation of such changes in long-lived plants, such as forest trees, while some attempts have been made in other organisms [11,12,49]. By using an experimental approach under different environmental conditions, we were able to analyze the phenotypic selection gradients and the correlation between female reproductive success and breeding values, as well as the genetic selection gradients and cross-environmental correlations at the seedling stage, when selection intensity is higher in natural forest tree populations.

Overall, our results suggest that significant genetic change is expected across generations in maritime pine and that those changes may strongly depend on local environmental conditions.

### Genetic control and environmental effects

Heritable transmission of traits across generations, a key component of evolutionary change [5], differed across environments, with much higher heritability under climatic chamber conditions than in the outdoor experiment –suggesting more potential for genetic change in less stressful environments. The only exception was for ontogenetic heteroblastic change (HON3), a trait that correlates with seedling field survival under drought in other pine species [50], and that was also involved in genetic selection gradients at seedling stage in our study (see below). Evolvability [10] was, however, much higher under outdoor semi-natural conditions, but also very variable across traits (see Table 1). Therefore, further studies on the potential for evolutionary change in forest trees need to include a wide range of traits and environments, especially as climatic models for trees predict different levels of regional maladaptation in the near future [51].

Despite its expected role as microevolutionary driver in heterogeneous environments [52], we did not find genetic variation for phenotypic plasticity (i.e. the treatment per family interaction) for any trait or treatment, either water regime or density. Lack of genetic differences in phenotypic plasticity for key adaptive traits, such as biomass allocation and survival, could be related to the cost of plasticity [52,53] that may limit its evolutionary importance in Mediterranean pine seedlings. However, one more likely explanation is the difficulty of the genotypes to track the optimum in complex and heterogeneous environments, as highlighted by the unexpected phenotypic changes in response to the treatments in our experiments: seedlings under the limited watering regime had lower root biomass allocation and higher height than under full water-capacity, while seedlings under the high density treatment had higher total biomass and height than under low density. Mediterranean environments are highly variable [54], and interactions among drought tolerance, intra- and inter-specific competition, and light use can result in variable life strategies among populations and individuals [55,56].

### Determinants of female effective reproductive success

Higher recruitment from the most successful mothers is mainly due to their seed quality. Significant and positive linear phenotypic selection gradients were detected at mature stage (parent trees) for the second principal component (PC2), mostly related to germination rate and number of empty seeds. Seed quality and parental phenotype are often correlated in plants, and a clear relation between mother-tree size, and seed output and quality exists [57], in agreement with sexual-selection theory predicting that female reproduction is limited by physical resources [58]. The phenotypic selection gradient for seed quality traits points also to a potential role of inbreeding. However, maritime pine is generally less affected by inbreeding than other pines in terms of general vigor [59], and often exhibits low inbreeding coefficients in natural populations, as estimated by molecular markers [60]. Despite phenotypic traits being reduced to independent PCAs, phenotypic selection gradients detected using Lande & Arnold’s [6] multivariate fitness analysis could be false-positives resulting from correlation with traits under selection that were not measured. However, this is unlikely in our study, as we included most of the adaptive traits that have shown differences for Mediterranean pines at the individual and population levels in several previous quantitative genetic studies [39,42-44].

The mass effect of seed quality on female effective reproductive success is modified by a distinct recruitment across families, seemingly derived from better adapted seedlings. Significant correlations among female relative reproductive success and breeding values (based on offspring performance) were detected for specific leaf area (SLA) under environment-controlled conditions and stem mass fraction (SMF) under semi-natural outdoor conditions, two adaptive traits related to abiotic stress-response in pines. The functional significance of these traits and their performance in the two environments provided insights into some potential mechanisms of evolutionary change in maritime pine. SLA was negatively correlated across the two experiments, indicating a different genetic basis. The lower SLA under controlled conditions for offspring from the mothers with higher reproductive success, could be related to the production of shorter needles with less transpirational surface area, while this same trait under the more stressful conditions of the semi-natural experiment may represent a largely plastic response to drought stress (as indicated by the lower heritability). SLA is related to the modulation of photosynthetic exchange (at equal levels of N in the needles [61]) and thus is a main component of relative growth rates. The adaptive importance of SLA under different stressful conditions is also well-known [62,63] and negative selection gradients for this trait have been found in other plants (e.g. [64]). With respect to SMF, changes in biomass allocation have been described as a strategy for drought tolerance in pine [38,44] and is correlated with fitness proxies (HTOT, see Methods) in genetic selection gradients under controlled conditions (see below). But under semi-natural conditions, despite significant genetic differences among families (see Table 1), the expected evolutionary potential is reduced due to the low genetic control of the trait (*h*^*2*^ of 0.041). Such small genetic effects are expected in natural environments [65].Variability in the correlation among reproductive success and functional or adaptive traits due to offspring’s environment can foster intra-population genetic diversity, even in the absence of spatial heterogeneity, as recruitment in forest trees depends largely on highly-variable annual environmental conditions [23].

### Genetic change and responses to selection

Genetic selection gradients (i.e. those based on genetic covariance between the trait and fitness) using seedling height as a fitness proxy indicated a different microevolutionary pattern between the two experimental conditions. Higher fitness is expected from plants with higher biomass allocation to stems (SMF, positive selection gradient) and a greater growth at the beginning of the growing season (*m* negative selection gradient) under (mild) environment-controlled conditions, but under more stressful outdoor conditions, seedlings with delayed ontogenetic development (HON3, positive selection gradient) will perform better. A strong negative correlation between height and survival in the outdoors experiment also suggests that a slow-growing drought-efficient phenotype is favored under stressful conditions. A bias in the estimation of selection gradients, which could have affected our estimates across the two environmental conditions [21], is unlikely, as we used a bivariate analysis [9], so these environment-dependent shifts in correlation among traits and fitness are rather the likely outcome of fluctuating selection in variable environments [66-68]. Low cross-environmental correlations in heterogeneous environments (see Additional file 1: Table S3) favor a more rapid approach to the optima and a more independent (less constrained) trait evolution [59].

The response to selection, *R*, associated with traits involved in significant genetic selection gradients (see above) was moderate when height was used as fitness proxy (~8-12%), but much higher when survival under semi-natural conditions was considered (~23%). In particular, ontogenetic change (HON3), a trait under relatively high genetic control (*h*^*2*^ of 0.276), appeared to be involved in microevolutionary change under these close-to-nature conditions. In Mediterranean environments, drought stress and inter-specific competition play overarching roles [23,55] and, therefore, individuals with a delayed developmental status (i.e. that reach a bigger size before ontogenetic changes take place) could be more efficiently established, as suggested by our results.

## Conclusions

Microevolutionary change due to differences in maternal reproductive success and early selection is expected in Mediterranean maritime pine populations, and could be an important mechanism mitigating the negative consequences of climate change. Substantial genetic variation was detected for adaptive traits related to growth, ontogenetic change and biomass allocation in maritime pine, but no clear differences in phenotypic plasticity among seedlings when two different factors (water availability and density) were considered. At mature-tree stages, higher female effective reproductive success can be mainly explained by a mass effect (seed quality), but was also modulated by the production of better-adapted seedlings. Selection gradients and responses to selection for seedlings differed across experimental conditions. Thus, although we have collected evidence of short-term genetic change for some relevant adaptive traits, directional changes are difficult to predict due to variable selection at different life stages and environments. In fact, contrasting experimental results highlight the relevance of testing genetic value under very different conditions to assess the possible trajectories of the response to selection. The distinct processes involved at the two life stages (mature trees or seedlings) together with environment-specific responses advise caution when predicting likely evolutionary responses to environmental change in Mediterranean forest trees.

### Availability of supporting data

The data sets supporting the results of this article are available in the LabArchives repository, doi:10.6070/H4JM27KW.

## Additional file

Additional file 1:
**Supporting Information.**

## Abbreviations

α: Significance level
*b*: Coefficient of the Gompertz function related to the maximum growth rate
CV_A_: Coefficient of additive genetic variation
COV: Covariance
D1: Outdoor experiment density (400 plants/m^2^)
D2: Outdoor experiment density (10,000 plants/m^2^)
DBH: Diameter (mother trees)
DBH10: Diameter increment in the last 10 years (mother trees).
DW: Total dry mass
*h*^*2*^: Narrow-sense heritability
HON3: Height at first production of axillary shoots
HT: Total height of the mother tree
HTOT: Total height of seedlings in experiments
LMF: Leaf mass fraction
*m*: Coefficient of the Gompertz function related to the inflection point
MOR: Mortality
NA: Not available
NE: Not estimable
ns: Not significant
NSU: Number of stem units
*R*: Response to selection
RMF: Root mass fraction
S.E: Standard error
SLA: Specific leaf area
SMF: Stem mass fraction
TRT: Treatment
W1: Watering regime at full-field capacity (45% in volume)
W2: Watering regime at 20% of full-field capacity (10% in volume)
WSEED: Seed weight (mg)
