# Supplementary material for: Environment-dependent microevolution in a Mediterranean pine (Pinus pinaster Aiton)
Source: BMC Evol Biol. 2014 Sep 23;14:200. doi: 10.1186/s12862-014-0200-5 (PMC4177426; doi:10.1186/s12862-014-0200-5)
Supplement: Additional file 1: — Supporting Information. [file 12862_2014_200_MOESM1_ESM.doc]

**Supporting Information**

Table S1. Morphological and reproductive traits of mother trees (*N*=22) and experiments in which their progenies were tested. *Experiment 1*: Environment-controlled conditions, *Experiment 2*: Outdoor semi-natural conditions.

| Tree ID | AGE  (yr) | HT  (dm) | DBH  (cm) | DBH10  (mm) | # Cones  (n) | Seed weight  (mg) | Germination rate  (%) | # of empty seeds  (%) | Reproductive success | Experiment |
| --- | --- | --- | --- | --- | --- | --- | --- | --- | --- | --- |
| PAA | 90 | 195 | 60.5 | 22 | 16 | 674.4 | 90 | 6 | 0.0942 | 1/2 |
| PA | 72 | 135 | 44.6 | 40 | 22 | 677.8 | 58 | 1 | 0.0078 | 2 |
| PB | 74 | 140 | 50.9 | 22 | 16 | 406.9 | 83 | 7 | 0.0355 | 1/2 |
| PD | 60 | 175 | 46.5 | 42 | 32 | 770.8 | 98 | 49 | 0.0269 | 1/2 |
| PE | 74 | 135 | 45.8 | 50 | 54 | 544.4 | 94 | 5 | 0.0286 | 1/2 |
| PF | 60 | 125 | 56.0 | 24 | 25 | 494.5 | 92 | 23 | 0.0886 | 2 |
| PG | 76 | 125 | 41.7 | 42 | 83 | 631.3 | 97 | 5 | 0.0606 | 1/2 |
| PH | 67 | 135 | 44.9 | 66 | 38 | 681.2 | 90 | 12 | 0.0622 | 1/2 |
| PI | 75 | 150 | 50.0 | 32 | 46 | 746.6 | 100 | 14 | 0.0597 | 1/2 |
| PJ | 65 | 140 | 36.3 | 44 | 36 | 501.3 | 91 | 4 | 0.0686 | 1/2 |
| PK | 72 | 132 | 39.2 | 68 | 26 | 541.8 | 82 | 5 | 0.0306 | 1/2 |
| PL | 62 | 115 | 43.0 | 18 | 18 | 554.9 | 82 | 5 | 0.0327 | 1/2 |
| PN | 84 | 140 | 43.6 | 36 | 0 | 523.2 | 88 | 25 | 0.0479 | 1/2 |
| PÑ | 59 | 130 | 39.5 | 66 | 51 | 630.3 | 90 | 18 | 0.0161 | 2 |
| PO | 82 | 135 | 46.5 | 34 | 20 | 564.5 | 73 | 3 | 0.0271 | 1/2 |
| PP | 77 | 140 | 45.5 | 70 | 46 | 683.0 | 14 | 77 | 0.0524 | 2 |
| PQ | 74 | 125 | 45.8 | 40 | 26 | 436.0 | 95 | 3 | 0.0342 | 1/2 |
| PR | 72 | 150 | 39.2 | 32 | 30 | 500.6 | 98 | 4 | 0.0349 | 1/2 |
| P053 | 62 | 127 | 35.0 | 40 | 20 | 752.1 | 100 | 47 | 0.0182 | 1/2 |
| P104 | 79 | 15.1 | 44.5 | 30 | 19 | 646.9 | 95 | 6 | 0.0885 | 1/2 |
| P129 | 60 | 124 | 29.6 | 38 | 31 | 833.1 | 89 | 7 | 0.0349 | 1/2 |
| P214 | 98 | 210 | 51.2 | 28 | 20 | 765.5 | 95 | 4 | 0.0500 | 1/2 |

AGE: age; HT: Total height; DBH: diameter of the tree at 1.30 m; DBH10: diameter increment in the last 10 years; Reproductive success: estimated by fractional parentage analysis based on successfully-established natural regeneration, after correction for distance effects between mother-trees and offspring (see Methods).

Table S2. Growth phases in environment-controlled conditions (*Experiment 1*).

| Phase | Duration  (weeks) | Watering treatments | Photoperiod  (day – night) | Temperature  (day – night) |
| --- | --- | --- | --- | --- |
| Acclimation phase | 3 | Full capacity | 12 – 12 | 20 ºC – 20 ºC |
| Growing phase | 8 | Full capacity | 16 – 8 | 25 ºC – 20 ºC |
| Growing phase | 12 | W1: full capacity or W2: 20% of full capacity | 16 – 8 | 25 ºC – 20ºC |
| Growing phase | 2 | Full capacity | 16 – 8 | 25 ºC – 20 ºC |
| Hardening phase | 2 | Full capacity | 12 – 12 | 25 ºC – 20 ºC |
|  | | | | |

Table S3. Correlation coefficients (and significance level) among breeding values based on offspring performance. Correlations for environment-controlled conditions (*Experiment 1*) are given under the diagonal, while those for the outdoor semi-natural experiment (*Experiment 2*) are above the diagonal. At the diagonal, the correlation coefficients across the two experiments are shown; ns: not significant; *: 0.01< α ≤0.05; **: 0.001< α ≤0.01; ***: α ≤ 0.001.

|  | HTOT | HON3 | NSU | DW | LMF | SMF | RMF | SUR | SLA |
| --- | --- | --- | --- | --- | --- | --- | --- | --- | --- |
| HTOT | 0.418 | 0.654 | 0.531 | 0.940 | -0.091 | -0.023 | 0.021 | -0.637 | -0.372 |
|  | * | ** | * | *** | ns | ns | ns | ** | ns |
| HON3 | 0.528 | -0.010 | -0.073 | 0.656 | -0.015 | -0.061 | 0.054 | -0.485 | -0.189 |
|  | ** | ns | ns | ** | ns | ns | ns | * | ns |
| NSU | 0.553 | -0.061 | 0.350 | 0.492 | 0.207 | -0.087 | -0.068 | -0.229 | -0.348 |
|  | * | ns | ns | * | ns | ns | ns | ns | ns |
| DW | 0.528 | 0.152 | 0.397 | 0.154 | -0.132 | -0.038 | 0.116 | -0.621 | -0.467 |
|  | * | ns | ns | ns | ns | ns | ns | ** | * |
| LMF | 0.294 | -0.282 | 0.540 | 0.155 | 0.088 | -0.526 | -0.636 | -0.066 | 0.492 |
|  | ns | ns | * | ns | ns | * | ** | ns | * |
| SMF | 0.836 | 0.684 | -0.591 | 0.462 | 0.138 | 0.027 | -0.308 | 0.078 | -0.105 |
|  | *** | ** | ** | ns | ns | ns | ns | ns | ns |
| RMF | -0.475 | 0.103 | -0.597 | -0.251 | -0.972 | -0.370 | 0.256 | 0.218 | -0.543 |
|  | ns | ns | * | ns | *** | ns | ns | ns | * |
| SUR | 0.694 | 0.379 | 0.496 | 0.135 | 0.418 | 0.425 | -0.499 | -0.192 | 0.202 |
|  | ** | ns | * | ns | ns | ns | * | ns | ns |
| SLA | 0.280 | 0.421 | 0.088 | -0.303 | -0.175 | 0.365 | 0.082 | 0.450 | -0.478 |
|  | ns | ns | ns | ns | ns | ns | ns | ns | * |

Table S4. (A) Correlation coefficients (and significance level) among morphological traits of the mother trees (*N*=22); ns: not significant; *: 0.01< α ≤0.05; **: 0.001< α ≤0.01; ***: α ≤ 0.001. (B) Principal component scores of phenotypic traits measured in mother trees.

**(A)**

|  | Age | Height | Diameter | DBH10 | # cones | Seed weight | # empty seeds | Germination  rate |
| --- | --- | --- | --- | --- | --- | --- | --- | --- |
| Relative fertility | 0.300 ns | 0.312 ns | 0.489  * | -0.271 ns | 0.001 ns | -0.031 ns | -0.054 ns | 0.131  ns |
| Age |  | 0.643 ** | 0.503  * | -0.235 ns | -0.215 ns | -0.003 ns | -0.212 ns | -0.109  ns |
| Height |  |  | 0.520  * | -0.249 ns | -0.222 ns | 0.358 ns | 0.012 ns | 0.135  ns |
| Diameter |  |  |  | -0.394 ns | -0.190 ns | -0.155 ns | -0.026 ns | -0.040  ns |
| DBH10 |  |  |  |  | 0.464 * | 0.136 ns | 0.362 ns | -0.376  ns |
| # cones |  |  |  |  |  | 0.162 ns | 0.066 ns | -0.004  ns |
| Seed Weight |  |  |  |  |  |  | 0.326 ns | -0.026  ns |
| # empty seeds |  |  |  |  |  |  |  | -0.479  * |

**(B)**

| Trait | PC1 | PC2 | PC3 |
| --- | --- | --- | --- |
| Age | 0.81291 | 0.00535 | 0.05016 |
| Height | 0.79789 | 0.10725 | 0.48385 |
| Diameter | 0.79016 | -0.02069 | -0.14047 |
| DBH10 | -0.49205 | -0.58327 | 0.25266 |
| # cones | -0.48923 | -0.09673 | 0.40507 |
| Seed weight | 0.03301 | -0.11097 | 0.89445 |
| # empty seeds | -0.04457 | -0.7677 | 0.27403 |
| Germination rate | -0.08874 | 0.89628 | 0.17531 |
| Eigenvalue | 2.4140 | 1.7665 | 1.3901 |
| Cumulative (%) | 0.3018 | 0.5226 | 0.6964 |
